# A Rare Case of a Malignant Proliferating Trichilemmal Tumor: A Molecular Study Harboring Potential Therapeutic Significance and a Review of Literature

**DOI:** 10.3390/dermatopathology11040038

**Published:** 2024-12-10

**Authors:** Mokhtar H. Abdelhammed, Hanna Siatecka, A. Hafeez Diwan, Christie J. Finch, Angela D. Haskins, David J. Hernandez, Ya Xu

**Affiliations:** 1 Department of Pathology & Immunology, Baylor College of Medicine, Houston, TX 77030, USA; 2 Department of Pathology & Laboratory Medicine, Ben Taub Hospital, Harris Health System, Houston, TX 77030, USA; 3 Department of Otolaryngology, Baylor College of Medicine, Houston, TX 77030, USA; 4 Pathology Service, HCA Houston Healthcare Clear Lake, 500 W. Medical Center Blvd., Webster, TX 77598, USA

**Keywords:** malignant proliferating trichilemmal tumors (MPTT), CD34, p53, HER2

## Abstract

Malignant proliferating trichilemmal tumors (MPTTs), arising from the external root sheath of hair follicles, are exceptionally rare, with limited documentation of their genetic alterations. We present a case of a 64-year-old African American woman who initially presented with a gradually enlarging nodule on her posterior scalp. An initial biopsy at an outside hospital suggested metastatic adenocarcinoma or squamous cell carcinoma (SCC) of an uncertain origin. A subsequent wide local excision revealed a 2.0 cm tumor demonstrating characteristic trichilemmal keratinization, characterized by an abrupt transition from the nucleated epithelium to a laminated keratinized layer, confirming MPTT. Immunohistochemistry demonstrated diffuse p53 expression, patchy CD 34 expression, focal HER2 membranous expression, and patchy p16 staining (negative HPV ISH). A molecular analysis identified TP53 mutation and amplifications in the ERBB2 (HER2), BRD4, and TYMS. Additional gene mutations of uncertain significance included HSPH1, ATM, PDCD1 (PD-1), BARD1, MSH3, LRP1B, KMT2C (MLL3), GNA11, and RUNX1. Assessments for the homologous recombination deficiency, PD-L1 expression, gene rearrangement, altered splicing, and DNA mismatch repair gene expression were negative. The confirmation of ERBB2 (HER2) amplification in the MPTT through a molecular analysis suggests potential therapeutic avenues involving anti-HER2 monoclonal antibodies. The presence of the TP53 mutation, without the concurrent gene mutations typically observed in SCC, significantly aided in this differential diagnosis.

## 1. Introduction

Malignant proliferating trichilemmal tumors (MPTTs) are uncommon malignant dermal neoplasms that arise from the external root sheath of hair follicles [1]. They typically manifest as solitary, painless masses on the scalps of elderly women, often presenting with ulceration, necrosis, or hemorrhage [1,2].

Under microscopic examination, the tumor displays a lobulated growth pattern with clusters of proliferating atypical epithelium showing trichilemmal differentiation, characterized by extensive trichilemmal keratinization and extension into adjacent tissues. MPTTs have the potential to exhibit aggressive behavior, including local invasion, recurrence, and metastasis. The tumor can be mistakenly diagnosed as squamous cell carcinoma (SCC). Distinguishing features, such as a history of slow tumor growth, the presence of trichilemmal keratinization, and findings from immunohistochemistry, are often used in the differential diagnosis.

The genetic alterations associated with MPTTs are poorly documented in the literature, which hinders the comprehensive characterization of their behavior, the prediction of clinical outcomes, and the development of targeted therapies. In this study, we present a case of MPTT with molecular analysis and provide a literature review to enhance the understanding of this rare condition.

## 2. Case Report

### 2.1. Clinical Presentation

**Figure 1:**
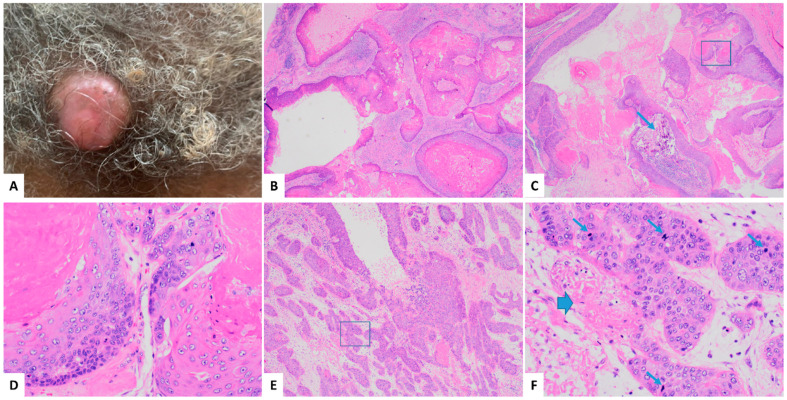
Clinical presentation and histologic examination of malignant proliferating trichilemmal tumors (MPTT) on a hematoxylin and eosin stain (H and E). The tumor presented as a 2.0 cm mass on the left occipital scalp (**A**). Microscopically, a histological examination revealed a solid and cystic dermal neoplasm, with smaller cystic spaces (**B**) 20X and a larger cyst exhibiting infolding bands of tumor cells with calcification indicated by an arrow (**C**) 20X. The tumor displayed an abrupt transition from the nucleated epithelium to a densely laminated keratinized layer without an intermediate granular layer (**D**) higher magnification of the squared area in (**C**) 200X. There were areas with invasive irregular tumor nests in the desmoplastic stroma, composed of nonkeratinizing tumor cells (**E**) 40X, showing moderate nuclear pleomorphism, frequent mitoses (indicated by narrow arrows), and occasional necrosis (indicated by wide arrow) (**F**) higher magnification of the squared area in (**E**) 200X.

The patient, a 64-year-old African American woman, noticed a slowly growing nodule on her posterior scalp over one year (Figure 1A). A biopsy conducted at another hospital initially suggested metastatic adenocarcinoma or squamous cell carcinoma (SCC) of an uncertain origin. She subsequently presented to our institution for further evaluation. A computed tomography (CT) scan revealed a 2.2 cm exophytic, partially calcified, heterogeneous soft tissue mass on the left occipital scalp, adjacent to the local left suboccipital musculature without bone invasion. The patient underwent wide local excision with 2 cm margins and limited lymph node dissection. Pathological examination confirmed MPTT with clear margins. Two months later, she completed a 5-week course of radiation therapy. At the 10-month follow-up, the patient remained disease-free.

### 2.2. Histopathologic Findings

The wide local excision specimen consisted of a skin ellipse measuring 6.5 × 6 × 1.5 cm, containing a central exophytic mass measuring 2 × 2 × 1.5 cm. The cut surface revealed a solid and cystic mass located 2.0 cm from the closest peripheral margin and 0.3 cm from the nearest deep margin. A histological examination revealed a solid and cystic dermal neoplasm (Figure 1B,C) showing characteristic trichilemmal keratinization, characterized by an abrupt transition from a nucleated epithelium to a densely laminated keratinized layer without an intermediate granular layer (Figure 1D). Areas with invasive tumor nests composed of nonkeratinizing tumor cells were observed (Figure 1E), displaying moderate nuclear pleomorphism, frequent mitoses, and occasional necrosis (Figure 1F). The findings of an invasive nonkeratinizing component in the tumor, characterized by nuclear pleomorphism, frequent mitoses, and necrosis, suggest a diagnosis of malignancy.

Immunohistochemistry (IHC) was performed using prediluted antibodies provided by the vendors. A standard antigen retrieval method was employed for the antibodies on the BenchMark Ultra automated IHC system. Specifically, Ventana’s Cell Conditioning Solution 1 (CC1), a pre-diluted Tris-based buffer provided by Ventana, was used as the antigen retrieval solution. This solution was applied at a high temperature (approximately 100 °C) on the automated slide stainer to effectively unmask the target antigens within the tissue prior to staining. Additional details about the antibodies are provided in Table 1.

**Figure 2:**
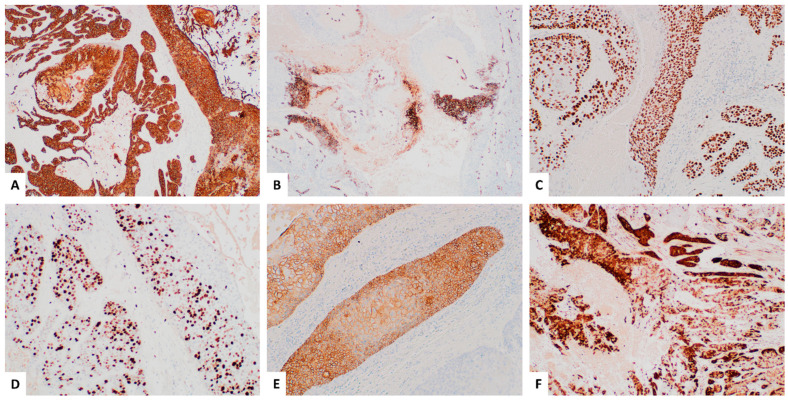
Immunohistochemistry (IHC) of the malignant proliferating trichilemmal tumor. IHC revealed diffuse expression of CK17 (**A**) 40X and p53 (**C**) 100X in the tumor, along with patchy positivity for CD34 (**B**) 40X. The Ki-67 proliferative index was approximately 30% in the hottest spots (**D**) 100X. Focal HER2 overexpression with a complete membranous staining pattern was observed (**E**) 100X. Additionally, patchy p16 staining was noted (**F**) 40X, whereas high risk HPV RNA in situ hybridization was negative.

The tumor demonstrated a diffuse membranous and cytoplasmic expression of CK17 (Figure 2A) and a diffuse nuclear expression of p53 (Figure 2C), along with patchy membranous positivity for CD34 (Figure 2B). The Ki-67 proliferative index was approximately 30% in the most active areas (Figure 2D). Focal HER2 overexpression, characterized by a complete membranous staining pattern, was assessed using breast cancer criteria (Figure 2E). HER2 FISH was positive. Additionally, focal/patchy p16 staining was observed (Figure 2F), while high risk HPV RNA in situ hybridization was negative. The diagnosis of MPTT was confirmed, with no evidence of lymph node involvement. 

### 2.3. Molecular Study

Molecular analysis was conducted as send-out tests, performed and interpreted by TEMPUS. The assay description from the report is as follows: “The Tempus xT (version 4) assay is a custom oncology testing panel consisting of 648 genes, with single nucleotide variants (SNVs), insertions and deletions (indels), copy number variants (CNVs), and chromosomal rearrangements (translocations) detected by hybrid capture next-generation sequencing (NGS) using custom-designed IDT probes.” The main genomic variants detected in the current case are summarized in Table 2.

NGS molecular analysis unveiled the genetic profile of the tumor, revealing a TP53 mutation (p.C176F missense variant) and amplifications in ERBB2 (HER2), BRD4, and TYMS. Additional gene mutations of uncertain significance included HSPH1, ATM, PDCD1 (PD-1), BARD1, MSH3, LRP1B, KMT2C (MLL3), GNA11, and RUNX1. Further evaluations for homologous recombination deficiency (HRD), PD-L1 expression, gene rearrangement, and altered splicing via RNA sequencing yielded negative results. Notably, there was no loss of expression detected in the DNA mismatch repair genes (MLH1, PMS2, MSH2, and MSH6).

## 3. Literature Review

A PubMed literature review spanning 24 years (from 2000, with expectations for a comparison of molecular results) summarized in Table 3, documented 41 studies on MPTTs of the scalp, involving 60 patients, including our case, aged 19–87 years (average of 57), with the majority being female (71.2%, 42/59; gender data missing in one study). The duration of MPTTs ranged widely from one month to 40 years, with the tumor sizes varying from 1.0 to 30.0 cm. A clinical follow-up was available for 78.3% (47/60) of the patients. The local recurrence rate was 21.7% (13/60). Metastases occurred in 23.3% (14/60) of the cases, involving lymph nodes and/or other sites, with the lymph nodes being the most common metastatic site (64.3%, 9/14 cases). The metastatic sites also included the brain, the base of the skull, the cistern sinus, the lung, the pleura, and the pancreas. The mortality rate was 11.7% (7/60). Notably, none of the previous reports included a molecular study.

## 4. Discussion

A previous literature review on MPTTs of the scalp reported an average patient age of 55 years, ranging from 26 to 85 years [2]. In our study of MPTTs of the scalp, the average age at presentation was 57 years, ranging from 19 to 87 years. The youngest MPTT patient, aged 19, had Keratitis–Ichthyosis–Deafness (KID) syndrome, a rare ectodermal dysplasia [8]. Females constituted the majority of the patients at 71.2%, consistent with previous studies [2,16]. The tumor sizes ranged from 1.0 to 30.0 cm in our study, compared to a range of 1–28 cm reported previously [2]. The duration of the MPTTs varied widely from 1 month to 40 years in our current study, whereas prior findings indicated a range of 1 to 30 years [16].

MPTTs of the scalp present a broad spectrum of differential diagnoses clinically, ranging from benign conditions such as trichilemmal cysts, pilomatricomas, and trichoepitheliomas to malignant entities like squamous cell carcinoma (SCC), basal cell carcinoma, and trichilemmal carcinoma. Clinically, MPTTs are most frequently mistaken for SCC. However, distinguishing MPTTs from SCC is critical, as MPTTs exhibit a significantly higher potential for local destruction and metastasis [2].

According to the WHO Classification of Tumors (5th edition, online version), MPTT falls within the morphological spectrum of PTT, which includes benign, atypical (intermediate), and rare malignant lesions [43]. MPTTs often arise from the transformation of a benign PTT and display aggressive clinical behavior and atypical histological features. Key differentiating characteristics include a rapidly enlarging mass, necrosis or hemorrhage, local invasion, or evidence of bone erosion on imaging. Early imaging studies, such as contrast-enhanced CT or, in some cases, magnetic resonance imaging (MRI), are essential for evaluating the extent of bone involvement and tumor invasion. Additionally, positron emission tomography (PET) can be employed to detect metastatic disease and assess the full extent of the tumor spread [2].

Histologically, MPTTs exhibit histopathologic features akin to a trichilemmal cyst, characterized by epithelial infoldings within cystic spaces [1].Tumor cells demonstrate abrupt differentiation towards large, monomorphic keratinocytes without a granular layer. Distinguishing MPTT from SCC can be challenging in areas with malignant features. The immunostains for CD34, p53, and Ki-67 have been documented to assist in MPTT diagnosis [16,18,20,33,44]. Four articles referenced the use of Ki-67 [9,20,28,39], with two reporting that MPTT demonstrated a 20% proliferative index labeled by Ki-67, supporting the diagnosis of malignancy [20,39]. We observed a proliferative index of 30% in the hottest spots. In our case, MPTT showed CK17 positivity, an epithelial marker. The tumor displayed focal or patchy CD34 immunoreactivity, suggesting trichilemmal differentiation from the outer root sheath, with most of the cells negative for CD34. Herrero et al. proposed that CD34 negativity could indicate tumor cell undifferentiation [43]. Therefore, CD34 expression aids in distinguishing MPTT from SCC. P53 overexpression and a high proliferative index labeled by Ki-67 confirm tumor malignancy, excluding diagnoses such as PTT and trichilemmal cysts. In this case, focal HER2 expression with complete membranous staining was noted. HER2 FISH was positive. Patchy but very focal strong p16 expression poses a diagnostic challenge, potentially leading to HPV-mediated SCC misdiagnosis in small biopsies.

Our molecular study identified amplifications in ERBB2 (HER2), BRD4 (Bromodomain protein 4), and TYMS (Thymidylate synthase). The ERBB2 amplification corroborates the IHC findings of HER2 overexpression, suggesting potential benefits of targeted HER2 therapies in MPTT using anti-HER2 monoclonal antibodies. BRD4, known for its frequent overexpression and role in drug resistance, has been proposed as a therapeutic target [45]. The amplification of TYMS, indicative of increased thymidylate synthase activity, may contribute to resistance against TYMS inhibitors, providing crucial insights for treatment planning [46].

In this case, a molecular analysis also detected a TP53 mutation, specifically the p.C176F missense variant located in exon 4 of TP53, characterized by a G to T substitution at nucleotide position 527. This mutation is distinct from previously reported mutations, such as the point mutation (CGT > CAT) at codon 273 in exon 8 of the TP53 gene observed in scalp MPTTs [44]. The presence of this TP53 mutation in MPTT, alongside the absence of the mutations commonly found in SCC such as CDKN2A, PIK3CA, KMT2D, and NOTCH1 [47], significantly contributed to the differential diagnosis.

Additional gene mutations of uncertain significance included HSPH1, ATM, PDCD1 (PD-1), BARD1, MSH3, LRP1B, KMT2C (MLL3), GNA11, and RUNX1. Although their specific roles in this tumor type are not fully understood, these mutations have the potential to influence the tumor’s pathogenesis and may affect its response to treatment.

Surgical management remains the cornerstone of treatment for MPTTs, complemented by radiotherapy and chemotherapy as adjuvant therapies in cases of aggressiveness or recurrence. Our study observed a local recurrence rate of 21.7%, consistent with the 24% reported in prior analyses [2]. Metastasis occurred in 23.3% of cases, predominantly affecting the lymph nodes, with additional sites including the brain, the base of the skull, the cistern sinus, the lung, pleura, and the pancreas, indicating both the lymphatic and hematogenous spread of MPTTs. The mortality rate was 11.7%, predominantly associated with cases showing recurrence and/or metastasis. Ye et al. established histologic criteria categorizing PPTs into three subgroups, facilitating the assessment of tumor behavior [8]. Benign PPTs often respond well to simple excision, while low-grade and high-grade MPTTs may necessitate more extensive surgical intervention and additional therapies [8]. Several chemotherapeutic regimens were used with limited success, with the CAV protocol (cisplatin, adriamycin, and vindesine) being the most commonly administered [2]. However, there is a potential risk of developing squamous cell carcinoma (SCC) due to malignant transformation induced by a high-dose chemotherapy regimen [2].

In summary, our study presents a case of MPTT of the scalp in an elderly woman, accompanied by a comprehensive literature review, detailed immunostain analysis, and genetic profiling. The identification of HER2 overexpression via IHC, confirmed by ERBB2 (HER2) amplification using FISH and NGS, suggests the potential therapeutic benefits of anti-HER2 monoclonal antibodies in treating aggressive MPTTs. The presence of TP53 mutation, distinct from the gene mutations typical of SCC, significantly aids in differential diagnosis. The amplification of BRD4 and TYMS, alongside mutations in other genes of uncertain significance, may contribute to understanding MPTT pathogenesis and guide future treatment strategies. Importantly, our molecular analysis of MPTT marks the first documented instance in the literature.

Although we present molecular analysis findings on MPTT, our data were derived from a single case, which limits the generalizability of the conclusions. Further molecular studies involving larger case series are essential to deepen our understanding of the molecular pathogenesis of MPTT. The mortality rate of this tumor, as previously mentioned, is 11.7%, emphasizing the clinical significance of addressing its aggressive behavior. Reducing the rates of recurrence and metastasis is crucial to improving patient outcomes and lowering the mortality associated with this tumor. A comprehensive molecular analysis may not only provide insights into the underlying mechanisms driving tumor progression but also identify potential therapeutic targets, paving the way for the development of targeted treatments.

## Figures and Tables

**Table 1:** Monoclonal antibodies utilized in immunohistochemical studies in the current MPTT case.

Antibody (Monoclonal)	CK17	P53	CD34	Ki-67	HER2	P16
Clone	SP95	Bp53-11	QBEnd/10	30-9	4B5	E6H4
Vender	Ventana /Roche	Ventana /Roche	Cell Marque	Ventana /Roche	Ventana /Roche	Ventana /Roche
Catalog #	790-4560	760-2542	134M-18	790-4286	790-7167	805-4713
Species	Rabbit	Mouse	Mouse	Rabbit	Rabbit	Mouse

#: Number.

**Table 2:** The main genomic variants detected in the current case of MPTT.

Clinical Summary	Gene	Alteration/Mutation Effect
Potentially Actionable	ERBB2 (HER2)	Copy number gain
Biologically Relevant	TP53	c.527G > T p.C176F NM_000546 Missense variant—loss of funcgtion (LOF)
	BRD4	Copy number gain
	TYMS	Copy number gain
Unknown Significance	HSPH1	c.2348G > T p.R783L Missense variant NM_001286504
	ATM	c.8938C > G p.L2980V Missense variant NM_000051
	PDCD1 (PD-1)	c.715G > A p.V239M Missense variant NM_005018
	BARD1	c.1339C > A p.L447I Missense variant NM_000465
	MSH3	c.177_178ins(18) p.A59_A60insPPAPPA Inframe insertion NM_002439
	LRP1B	c.10414G > A p.D3472N Splice region variant NM_018557
	KMT2C (MLL3) c	c.7443-2dup Splice region variant NM_170606
	GNA11	c.735+1_736-1del Splice region variant NM_002067
	RUNX1	c.1265A > C p.E422A Missense variant NM_001754

**Table 3:** Literature review of 41 studies on malignant proliferating trichilemmal tumor of the scalp covering 60 cases, including the current study, over the past 24 years (2000–2024) in PubMed.

Year [Ref #]	Age	Case # /Sex (F/M)	Duration (mo, y)	Size (cm)	Local Recur (#)	Metastasis /Site (#)	F-U (mo, y) (#)
2000 [3]	61	1/F	20 y	16.0	NA	Brain	12 mo/died
2001 [4,5]	51	1/F	6 y	1.2	NA	None	NA
	32	1/M	1 y	8.0	None	LN, Brain	6 mo
2002 [6]	60	1/F	4 y	5.0	NA	None	L to F-U
2003 [7]	69	1/F	2 y	2.0	Recur	LN	8 mo
2004 [8]	41–87	11/F, 3/M	1 mo–20 y	1.0–9.0	Recur (3)	LN (1)	3 mo–8.5 y Died (2) Ϯ
2005 [9]	33	1/M	1 y	10.0	None	None	~1.5 y
2006 [10]	54	1/F	3 y	3.0	None	None	2 y
2007 [11,12]	32	1/F	NA	12	Recur	LN	4 mo
	19	1/M	NA	5.0	Recur	BOS, CS, Lung	5 y/died
	50	1F	3 mo	6.7	NA	LN	NA
2008 [13,14,15]	72	1F	10 y	2.0 (recur)	Recur	None	32 mo
	32	1M	1 y	2.5	None	LN	9 mo/died Ϯ
	76	1F	Recent	2.0	None	None	11 mo
2009 [16,17]	85	1F	1 y	15.0	None	None	14 mo
	58	1F	2 mo	5.5	NA	None	L to F-U
2010 [18,19]	58	1F	1 y	3.2	NA	None	NA
	41	1F	1 y	NA	NA	None	NA
	51	1F	NA	16	Recur	None	54 mo
	53	1M	Rapid	20	Recur	Brain	9 mo/died
2011 [20,21]	65	1F	9 y	2.0	None	None	6 mo
	57	1F	Many y	7.0	NA	None	L to F-U
2012 [22,23]	73	1F	3 y	6.0	NA	None	L to F-U
	25	1F	1 y	4.0	None	None	1 y
2013 [24]	62	1F	NA	NA	None	Left pleura	3 y
2014 [25,26,27]	65	1F	4 mo	3.0	NA	None	NA
	32	1M	8 mo	15.0	NA	None	F-U
	26	1F	1 y	6.0	Recur	LN	1 mo
2015 [28,29,30]	67	1M	3 mo	3.0	None	None	6 mo
	48	1F	NA	4.0 (recur)	Recur	None	~2 y
	65	1M	40 y	NA	None	LN, Pancreas	15 mo
2016 [31,32,33]	61	1M	15 y	15.0	NA	None	L to F-U
	29	1M	7 y	30.0	NA	Lung	2 mo/died
	42	1M	11 y	22.0	Recur	None	28 mo
2017 [34]	64	1F	18 mo	12.0	None	None	3 mo
2018 [35]	56	1F	1 y	1.8	Recur	None	6 mo
2019 [36]	68	1F	20 y	~15.0	NA	None	NA
2020 [37]	Old	2F	NA	NA	None	None	2 y
2021 [38]	46	1F	Many y	1.5	NA	None	F-U
2022 [39,40]	85	1M	2 y	6.8	None	None	7 mo
	69	1M	1 y	2.0	Recur	LN, Lung	26 mo/died
	55	1F	1.5 y	11.0	None	None	10 mo
	NA	1 (NA)	NA	10.0	Recur	None	2 mo/died
2023 [41]	86	1F	Several y	4.0	None	None	2 y
2024 [42]	87	1M	NA	6.0	None	None	6 mo
Current c	64	1F	1 y	2.0	None	None	10 mo

**Ref: reference; #: number of cases; F: female; M: male; mo: month(s); y: year(s); cm: centimeter(s); NA: not available; LN: lymph node; BOS: base of skull; CS: cistern sinus; L to F-U: lost to follow-up; C: case**. Ϯ *Total two patients died of diseases other than MPTT.*

## Data Availability

The original contributions presented in this study are included in the article; further inquiries can be directed to the corresponding authors.
